# Highly Unidirectional Radiation Enhancement Based on a Hybrid Multilayer Dimer

**DOI:** 10.3390/nano12040710

**Published:** 2022-02-21

**Authors:** Dengchao Huang, Shilin Liu, Kang Yang

**Affiliations:** Key Laboratory of Advanced Perception and Intelligent Control of High-End Equipment, Ministry of Education, College of Electrical Engineering, Anhui Polytechnic University, Wuhu 241000, China; huangdengchao19@163.com (D.H.); sl.liu@ahpu.edu.cn (S.L.)

**Keywords:** hybrid nanoantenna, unidirectional emission, metal–dielectric–metal dimer

## Abstract

Dimers made of plasmonic particles support strong field enhancements but suffer from large absorption losses, while low-loss dielectric dimers are limited by relatively weak optical confinement. Hybrid dimers could utilize the advantages of both worlds. Here, we propose a hybrid nanoantenna that contains a dimer of core-dual shell nanoparticles known as the metal–dielectric–metal (MDM) structure. We discovered that the hybrid dimer sustained unidirectional forward scattering, which resulted in a nearly ideal Kerker condition in the frequency close to the resonance peak of the dimer due to enhancing the amplitude of the induced high-order electric multiples in the gap and effectively superimposing them with magnetic ones, which respond to the excitation of the plane wave in the dielectric layer of the dimer. Furthermore, when an electric quantum emitter is coupled to the dimer, our study shows that the optimal hybrid dimer simultaneously possesses high radiation directivity and low-loss features, which illustrates a back-to-front ratio of radiation 53 times higher than that of the pure dielectric dimer and an average radiation efficiency 80% higher than that of the pure metallic dimer. In addition, the unique structures of the hybrid hexamer direct almost decrease 75% of the radiation beamwidth, hence heightening the directivity of the nanoantenna based on a hybrid dimer.

## 1. Introduction

Nanoantennas and resonant nanoparticles have become essential building blocks for controlling and engineering light’s electromagnetic behavior at subwavelength scales, with numerous applications in optical and quantum communications, sophisticated manipulations of quantum dot emissions, optical polarization, and optical forces [1,2,3,4,5]. Among these metrics, dimer nanoantennas have received widespread attention due to their unique optical properties in transmitting and controlling the energy radiated by a quantum emitter which is suitable for the creation of an efficient single-photon source [6,7]. Furthermore, the nanoantenna may flexibly modify the resonant wavelength by managing the gap width of the dimer, enabling exceptional tunability across a large optical spectral range [8,9].

Up to now, the metal [10,11] (e.g., Ag, Au) and high-permittivity dielectric dimer (e.g., silicon) [12,13] have been successfully applied in many kinds of platforms. For the metallic dimer, each elementary component has the ability to manifest the free electron oscillation near the surface of the metal (so-called surface plasmon resonance (SPR)). These resonances can couple with each other in the gap, creating a hybridized mode depending on the incident light polarization. However, these hybridizations approve a strong field enhancement “hot-spot” in the gap region, plasmonic excitations are known to be affected by relatively parasitic large absorption loss which remarkably increases the probability of nonradiative photon energy loss and may be unfavorable for applications such as quantum emitter radiation [12,13]. This unavoidable problem has stimulated the study of dielectric counterparts with high permittivity and low loss, where a dielectric dimer can support control of the near-field enhancement and far-field radiation properties of nearby emitters through the generation of displacement current induced inside the nanostructure [14,15]. In comparison to a metallic dimer, the dielectric dimer has the advantage of low absorption loss. However, the dielectric dimer’s field enhancing capabilities are fairly weaker which restrain its applications such as Single Molecule Detection [16], quantum emitter enhancement [17], and SERS [18].

In order to simultaneously fulfill both high far/near low-loss features and field improvement, it is general to construct a hybrid materials (metal/dielectric) dimer that could combine both advantages of the metal and dielectric. Recently, some researchers attempted to create this by mixing various metallic and dielectric nanostructures such as metal–dielectric waveguide [19], hybrid metal–dielectric Yagi-Uda nanoantenna [20], an asymmetric dimer containing a small metal particle and large dielectric nanosphere [21], and so on. However, these hybrid nanostructures only show excellent performance in some specific solutions (e.g., hybrid metal–dielectric nanoantenna can sustain a high radiation ratio of forward-to-back (49.2) in the wavelength 600 nm depending on the specific quantum emitter [20]), and the underlying coupling mechanism has not yet been discovered. Furthermore, most of the works only focused on the dimer with pure metal or dielectric elementary components showing dipole–dipole resonances without a strong overlap between the magnetic and electric high-order responses. When the two-component hybrid materials (e.g., metal–dielectric–metal nanostructure) are incorporated together, the overall optical spectral response of the hybrid dimer would be controlled by the interaction between the metal SPR and the different dielectric mixed high-order resonances and the researcher presents a reconfigurable hybrid metasurface platform by incorporating the phase-change material Ge_2_Sb_2_Te_5_ (GST) into metal–dielectric meta-atoms for active and non-volatile tuning of properties of light [22]. However, there are few studies revealing the coupling mechanism of the metal–dielectric–metal hybrid dimer.

In this work, first, we reveal the mechanism of unidirectional forward scattering in the symmetric metal–dielectric–metal dimer under a plane wave. Under a specific polarized wave plan, the symmetric dimer spheres sustained unidirectional forward scattering with a high forward-to-backward radiation ratio (almost 40), which resulted in a nearly ideal Kerker condition in the frequency close to the resonance peak of the dimer. All the exhibited features in the spectrum are analyzed via the multipole decomposition expansion to determine the relative contributions of different electric and magnetic multipolar resonances [23,24,25]. After that, we examine the corresponding back-to-front ratio of radiation to disclose how an electric dipole emitter interacts with a hybrid dimer. The optimized symmetric MDM dimer manifests a high backward-to-forward radiation ratio (surpassing 53 and almost attaining the second Kerker condition) at 814 THz with a high radiation efficiency of 87% according to a flexible position of the emitter and the distance of gap. Finally, our simulation experiments compare the far-field radiation properties of the emission enhancement of the hybrid MDM dimer with a pure metallic dimer and other hybrid counterparts. The hybrid MDM dimer nanoantenna shows better unidirectional radiation performance (such as F/B ratio of 40 and B/F ratio of 53) under the wave plane and dipole emitter, respectively. We observed that the optimal MDM trimer, tetramer, and hexamer can also sustain unidirectional forward scattering with high scattering efficiency and extremely small beamwidth (below 6.9 deg). These results would offer an initial assessment of the potential of the hybrid MDM dimer antenna on light directivity manipulation and quantum emitter radiation enhancement.

## 2. Materials and Methods

The far-field radiation patterns of the hybrid dimer nanoantenna were calculated using the three-dimensional finite-difference time-domain method (CST Microwave Studio Environment 2018) [26]. In the simulated FDTD calculations, a point dipole source representing a single emitter was used. The far-field angular patterns of the emitter dimer in the free space were acquired by capturing the field components in closed box monitors. 

We performed a multipole decomposition expansion based on the induced multipole moments to gain a better understanding of the optical property of the hybrid dimer nanoantenna [27,28]. In order to determine the scattered field for the hybrid dimer in air, a plane wave was utilized as the excitation source with electric polarization perpendicular to the dimer axis and with electric polarization perpendicular to the dimer axis. The total scattering response of the nanoantenna for the plane wave excitation, according to [29]:

(1) $$C_{sca}=\frac{\pi }{k^{2}}\sum_{l=1}^{\infty }\sum_{m=-l}^{l}\left(2l+1)({\left|a_{E}(l,m)\right|}^{2}+{\left|b_{M}(l,m)\right|}^{2}\right)$$

here, $a_{E}(l,m)$ and $b_{M}(l,m)$ are the multipole coefficients of electric and magnetic, respectively. They can be expressed as:

(2) $$a_{E}\left(l,m\right)\frac{{\left(-i\right)}^{l-1}k^{2}\eta O_{lm}}{E_{0}{\left[\pi \left(2l+1\right)\right]}^{\frac{1}{2}}}=\exp\int \left(-im\phi \right)\{\left[\Psi_{l}\left(kr\right)+\Psi_{l}^{\prime \prime }\left(kr\right)\right]P_{l}^{m}\left(\cos\theta \right)\hat{r}\cdot J_{S,j}\left(r\right)\;+\frac{\Psi_{l}^{\prime }\left(kr\right)}{kr}\left[\left(\theta \right)\hat{\theta }\cdot J_{S,j}\left(r\right)-i\pi_{lm}\left(\theta \right)\hat{\phi }\cdot J_{S,j}\left(r\right)\right]\}d^{3}r$$

(3) $$a_{M}\left(l,m\right)=\frac{{\left(-i\right)}^{l+1}k^{2}\eta O_{lm}}{E_{0}{\left[\pi \left(2l+1\right)\right]}^{1/2}}\int \exp\left(-im\phi \right)j_{l}\left(kr\right)\left[i\pi_{lm}\left(\theta \right)\hat{\theta }\cdot J_{S,j}\left(r\right)+\tau_{lm}\left(\theta \right)\hat{\phi }\cdot J_{S,j}\left(r\right)\right]d^{3}r$$

where $\psi_{l}(kr)=krj_{l}(kr)$ are the Riccati–Bessel functions, ${\psi \prime }_{l}(kr)$ and ${\psi \prime \prime }_{l}(kr)$ are their first and second derivatives and other functions $O_{lm}=\frac{1}{{\left[l\left(l+1\right)\right]}^{\frac{1}{2}}}{\left[\frac{2l+1}{4\pi }\frac{\left(l-m\right)!}{\left(l+m\right)!}\right]}^{\frac{1}{2}}\;$. $\tau_{lm}\left(\theta \right)=\frac{d}{d\theta }P_{l}^{m}\left(\cos\theta \right)$ and $\pi_{lm}\left(\theta \right)=\frac{m}{\sin\theta }P_{l}^{m}\left(\cos\theta \right)$ are associated as the Legendre polynomials $P_{l}^{m}$. These $a_{E}(l,m)$ coefficients and $b_{M}(l,m)$ determine the electric and magnetic multipoles, namely dipole, quadrupole, octupole, hexadecapole, dotriacontapole, and so on. The electric dipole, hexadecapole, dotriacontapole, and magnetic octupole, hexadecapole are primarily contributed to the extinction spectra. Other coefficients are thus neglected since these contributions to the scattering were very low in this study. We want to design a hybrid dimer nanoantenna that can simultaneously control unidirectional radiation of emitter and incident light. For illustration purposes, Ag and Ge are used as metal and dielectric materials, respectively, while other metal (e.g., Au, Al, etc.) or dielectric (e.g., GaP, GaAs, etc.) materials can also be adopted. In this study, we used high-permittivity germanium (Ge) as the nanoparticle’s intermediate layer due to low dissipative loss in the work spectrum. The optimized coupled MDM symmetric dimer consists of two Ag–Ge–Ag multilayer nanoparticles with diameters (25 nm, 100 nm, 230 nm). The optical properties of Ag and Ge were obtained from Palik [30,31] and the fabrication of the hybrid dimer nanoantenna can be used by the approach of [32]. It reported a novel approach (seed-mediated growth method and self-assembling) for fabricating core-shell 3D nanoparticles. Furthermore, it can control the gap of dimer by using the properties of polyethyleneimine. Finally, we would get the hybrid MDM dimer nanoantenna with different gaps and the experimental data of the property of hybrid dimer nanoantenna can be collected by near-field scanning optical microscopy (NSOM) [33].

## 3. Results

**Figure 1:**
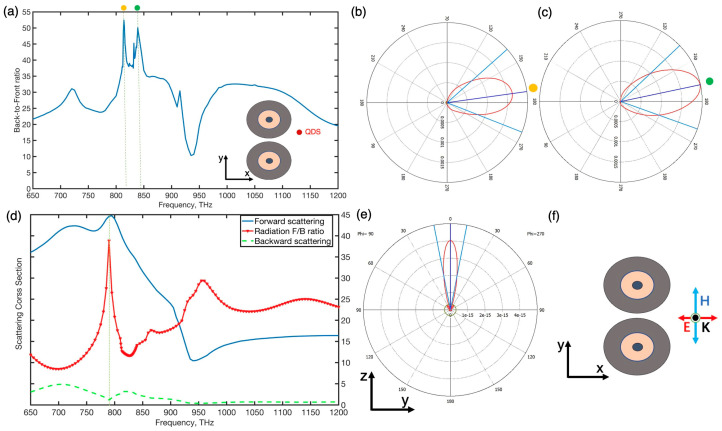
(**a**) The optimized symmetric MDM dimer which is excited by quantum dots source manifests two peaks of the backward-to-forward radiation ratio (surpass 53 and 50 almost attain the second Kerker condition) at 814 THz and 845 THz, respectively. (**b**,**c**) insect 814 THz and 845 THz radiation pattern in the x–y plane, respectively. (**d**) Under (polarization parallel to the dimer axis) specific polarized wave plane, the symmetric dimer spheres sustained unidirectional forward scattering which results in a nearly ideal Kerker condition in the frequency 795 THz close to the resonance peak of the dimer. (**e**,**f**) insect 795 THz radiation pattern in the y–z plane and the MDM dimer nanoantenna under incident light (polarization parallel to the dimer axis) in the x–y plane.

**Figure 2:**
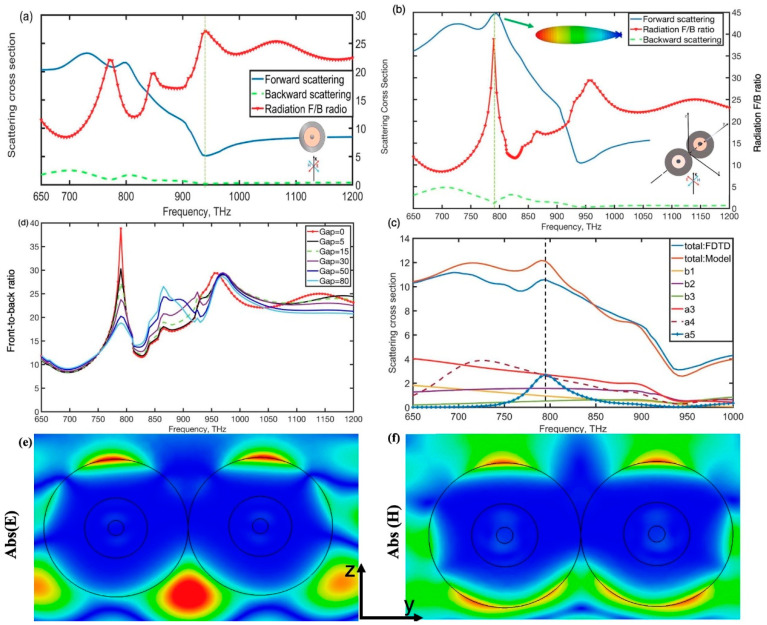
(**a**) the 230 nm diameter single MDM sphere and (**b**) the dimer of these coupled spheres scattering cross-section in the forward and backward directions, as well as the F/B ratio, (**c**) for a gap separation of 0 nm, numerical simulations of scattering spectra of coupling situations were compared to FDTD results and (**d**) the F/B ratio of the dimer with different gaps, in addition, radiation pattern diagrams at a certain frequency are shown in the inset. (**e**,**f**) the near-electric and near-magnetic field in the y–z plane at the frequency of 795 THz.

We first investigated the properties of the single hybrid sphere and the hybrid dimer nanoantenna consisting of two identical spheres under a plane wave for electric polarization parallel to the *x*-axis, as shown in Figure 1. The structural characteristics of these two MDM spheres are 25 nm–100 nm–230 nm (antenna gap width = 0 nm), are coupled in air and form the hybrid dimer nanoantenna. Because of the generation of intense displacement currents in the dielectric layer and the collective polarization of conduction electrons in metal layers, the individual MDM nanoparticles can be turned to support both electric and magnetic dipolar or multipolar optical responses. In the F/B ratio spectra of the single hybrid sphere, as shown in Figure 2a, several peaks were observed at frequencies in which the general Keker condition was achieved. However, the scattering efficiency at those frequencies was low owing to the Kerker condition that occurs away from the resonance peaks. When the MDM nanoparticles are coupled together to constitute the dimer, they can support more coupled electric and magnetic multiple modes than the single nanoparticle and they offer field enhancement and confinement of electromagnetic energy in the gap. Furthermore, the MDM dimer nanoantenna supports high forward scattering intensity and narrow beamwidth due to effectively induced multiple modes. The coupled hybrid dimer showed a new distinct peak in its F/B ratio spectra at around 795 THz, at which the forward scattering attains a peak and backward scattering was suppressed, and compared to the individual particle, the hybrid dimer can almost achieve the first Kerker condition (almost zero backward scatterings) in the frequency close to the resonance peak since the electric hexadecapole resonance in the particle was effectively excited at around 795 THz [34]. The theoretical analysis of scattering spectra of the coupling between the MDM dimer and their multipole decomposition results for partial electric and magnetic multipoles is shown in Figure 2c. As shown in Figure 2c, these two distinctly forward scattering cross-section peaks of the hybrid dimer are observed around 720 THz and 795 THz in the scattering spectra, which correspond to the coupling of induced electric hexadecapole and dotriacontapole resonances, respectively. The high-order magnetic resonance, which responds to the excitation of the plane wave in the dimer, may help improve directivity and restrict the radiation beam [35]. The dimer’s scattering cross-section in both the forward and backward directions with different gaps was also observed in this section. From Figure 2d, we found that the distance of the gap would greatly affect the values of the scattering F/B ratio due to a small gap of dimer effectively enhancing the magnitude of electric high-order moments formed in the gap. Figure 2e,f show the near-electric and near-magnetic field in the y–z plane at the frequency of 795 THz. It indicated that high-order electric resonances were effectively excited in the dimer gap at 795 THz and the hybrid dimer nanoantenna induced stronger high-order magnetic modes than the dipole magnetic mode, as shown in Figure 2f. In addition, these electric high-order multiples superimpose magnetic ones induced in the dimer can contribute to improving the directivity.

**Figure 3:**
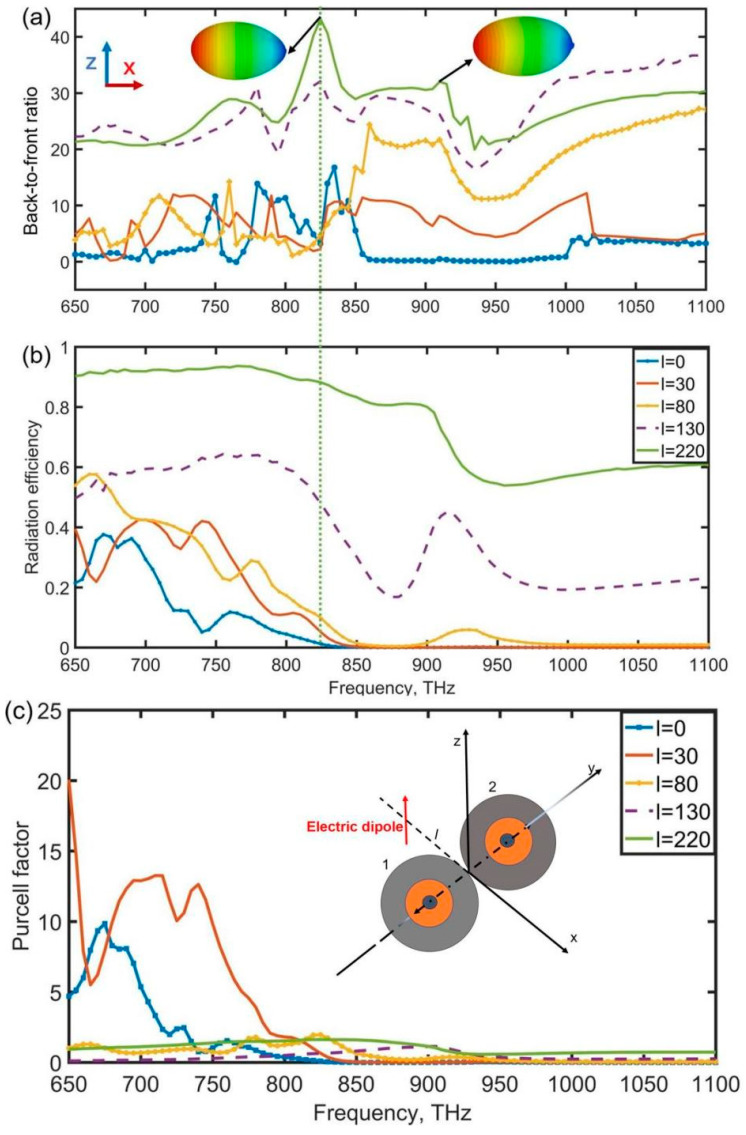
Back-to-front ratio (**a**), radiation efficiency (**b**), and Purcell factor (**c**), of the symmetrical dimer which is excited by a point-like electric dipole source as a function of the distance between the coupled antenna and the electric dipole source, and (**a**) insect radiation patterns in the x–y plane of frequency 835 THz and 910 THz, respectively.

In optics, quantum dots and other quantum sources are inefficient as light sources as their impedances do not match the open space impedance. Besides the improvement emission efficiency of quantum sources, another important problem is how to efficiently capture the energy emitted by a single quantum emitter. In general, a single quantum source radiates along the omnidirectional in the free space. However, in the optical frequency range, nanoantennas present unique structures such that the decay properties of a single emitter are enhanced. Nanoantennas are used to efficiently match the impedance of a quantum source to free space, resulting in a significant increase in radiation efficiency. An emitter located around the gap is modified due to two processes: the emitter transfers the energy to the nanoantenna and the coupling field to the outgoing radiation by the emitter [36]. Therefore, these nanoantennas have the ability to confine strong electric near-field in a region and to control the radiation of the emitter in the desired direction. When the independent MDM nanoparticle is excited by a plane wave, it can be turned to sustain both electric and magnetic dipolar or multipolar responses resulting in the generation of intense displacement currents in the dielectric layer and the collective polarization of conduction electrons in the metal layers. However, the individual nanoparticle only dominates an emitter unidirectional radiation relies on a specific incident dipole source [37,38]. Therefore, we utilize the MDM symmetric nanoparticle to form the dimer, they can support more coupled electric and magnetic multiple modes than the single nanoparticle for various emitters. They also offer field enhancements, electromagnetic energy confinement in the gap and high forward scattering efficiency with narrow beams according to effectively induced multipole modes. Furthermore, each sphere’s construction allows it to preserve its resonances in the spectrum’s low absorption spectrum [39]. For these purposes, we used the hybrid dimer nanoantenna that can simultaneously support metal SPR and different dielectric mixed high-order resonances which result in strong electric near-field and unidirectional radiation in the nanostructure.

(4) $$B/F=\frac{\int_{\theta_{c}-\delta }^{\theta_{c}+\delta }\int_{\pi -\delta }^{\pi +\delta }S(\varphi ,\theta )\sin\theta d\theta d\varphi }{\int_{\theta_{c}-\delta }^{\theta_{c}+\delta }\int_{-\delta }^{\delta }S(\varphi ,\theta )\sin\theta d\theta d\varphi }$$

where S(φ,θ) is the intensity of the back focal plane with an azimuthal angle (φ) and polar angle (θ). Figure 3a shows the characteristics of three-dimensional far-field intensity distribution and that of the 2D directional properties of the hybrid system on the y = 0, x–z plane. As shown in Figure 3a, the back-to-front ratio of the hybrid system increases as the distance between the dimer and the point-like electric dipole source increases, and the B/F ratio has peaked at f = 835 THz and f = 910 THz which attain 43 and 30, respectively. Insets show the three-dimensional radiation patterns of the structures at these frequencies. It demonstrates that broadband near-zero-forward radiation (second Kerker condition) can be achieved by optimizing the source location since contributions from magnetic and electric high-order Mie components are included. In comparison to the first Kerker condition, which requires identical electric and magnetic multipole coefficients (a_1_ = b_1_) [40], the interaction between electric and magnetic high-order coefficients created in the gap with varying amplitudes provides the basis for our suggested mechanism.

The high back-to-front ratio is often associated with a significant increase in dissipative losses [35]. The great radiation efficiency of this nanoantenna is achieved by decreasing the power dissipated in the nanoantenna losses. When mainly electric multiple moments are excited in a nanoantenna, it leads to a dominance of the strong electric near-field over the magnetic one, resulting in more power losses. The reason is the dielectric material has no dissipated magnetic energy. As shown in Figure 3b, when the electric dipole is close to the nanoantenna, the radiation efficiency decreases rapidly due to the high near-electric field causing high power losses. On the contrary, when the electric dipole keeps away from the nanoantenna, the radiation efficiency increases effectively. The radiation efficiency (l = 220 nm) attains 80% in the frequency range of 650 THz~900 THz where the back-to-front ratio exceeds 20. Especially, the back-to-front ratio has a peak of 43 at f = 835 THz with a radiation efficiency of 87%, as shown in Figure 3a. Figure 3a also shows radiation patterns in the x–y plane of frequencies at 835 THz and 910 THz. It is clear to see that the hybrid dimer nanoantenna has the ability of unidirectional radiation under the excitation of a dipole source. The Purcell factor is also discussed in this paper which can be expressed as:

(5) $$F_{rad}=\left(\frac{P_{rad}+P_{nonrad}}{P_{0,rad}}\right)\times \eta_{rad}$$

Here, $P_{rad}$ is the power radiated in the far zone (enhanced by the nanoantenna), $P_{nonrad}$ is the power dissipated in the environment (contain the power loss in the nanoantenna), and $P_{0,rad}$ is the power radiated in free space by the same emitter. The radiation efficiency of the nanoantenna is determined as follows:

(6) $$\eta_{rad}=\frac{P_{rad}}{P_{rad}+P_{nonrad}}$$

The relationship between the Purcell factor of the hybrid dimer nanoantenna and the distance parameter l can be seen in Figure 3c. When the distance is below 80 nm, many peaks can attain 20, 10, and 13 at around 650 THz, 670 THz, and 750 THz, respectively. As the distance is larger than 80 nm, the Purcell factor decreases rapidly due to the weakening interaction between the point-like electric dipole and the coupled antenna.

**Figure 4:**
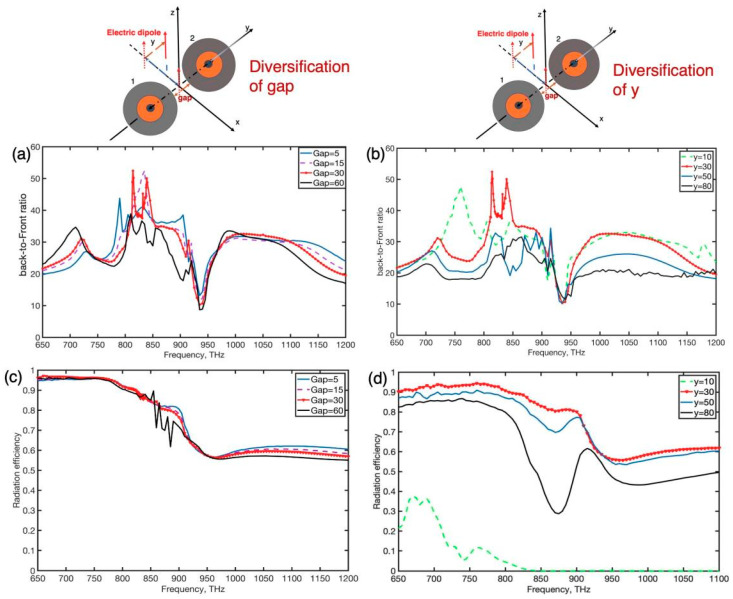
(**a**,**b**) back-to-front ratio, and (**c**,**d**) radiation efficiency, of the symmetrical dimer which is excited by a point-like electric dipole source as a function of the bandwidth of the gap in the dimer and y coordinate of the emitter with fixed l = 220 nm, respectively.

Like other dimer nanoantennas [41,42], the coupling strength between the MDM spheres and metal and dielectric constituents of the dimer depends greatly on the gap distance and the position of the emitter. Thus, as illustrated in Figure 4, we may measure optical responses of the dimer with a variety of gap sizes and varied displacements (along the *y*-axis) of the emitter. In all of these instances, the dipole emitter was situated at 220 nm distance from the center of the dimer (l = 220 nm) to maximize the efficiency of the dipole. As the gap increased, the radiation efficiency remained unchanged, but the optimal B/F ratio occurred at f = 814 THz, f = 835 THz, and f = 839 THz which attain 53, 50 and 51, respectively. When the gap was 60 nm, the strong directivity (B/F > 40) was even preserved. As a result, a huge working gap can achieve a unidirectional effect. We also discovered that far-field emission features were sensitive to the displacement of the emitter position in the y-direction, as shown in Figure 4b,d. As the distance was increased with the positive y-direction, the radiation efficiency obviously increased due to the phase and magnitude of magnetic and electric high-order induced in the gap fulfilled the second Kerker condition. As a result, the emission forwards are almost near zero and the non-radiative loss is lower. When the displacement was 30 nm, the optimal B/F radio occurred at 814 THz and 835 THz, the emission main lobe was observed to be shifted to 15 deg as displayed in Figure 1. With the further enlargement of the offset to 80 nm, the intensitive property of unidirectional emission (B/F > 30) was attained. Such beam radiation characteristics are promising for applications such as optical communications. This hybrid dimer nanoantenna’s enhanced unidirectional radiation might be suitable for an oriented quantum light source.

**Figure 5:**
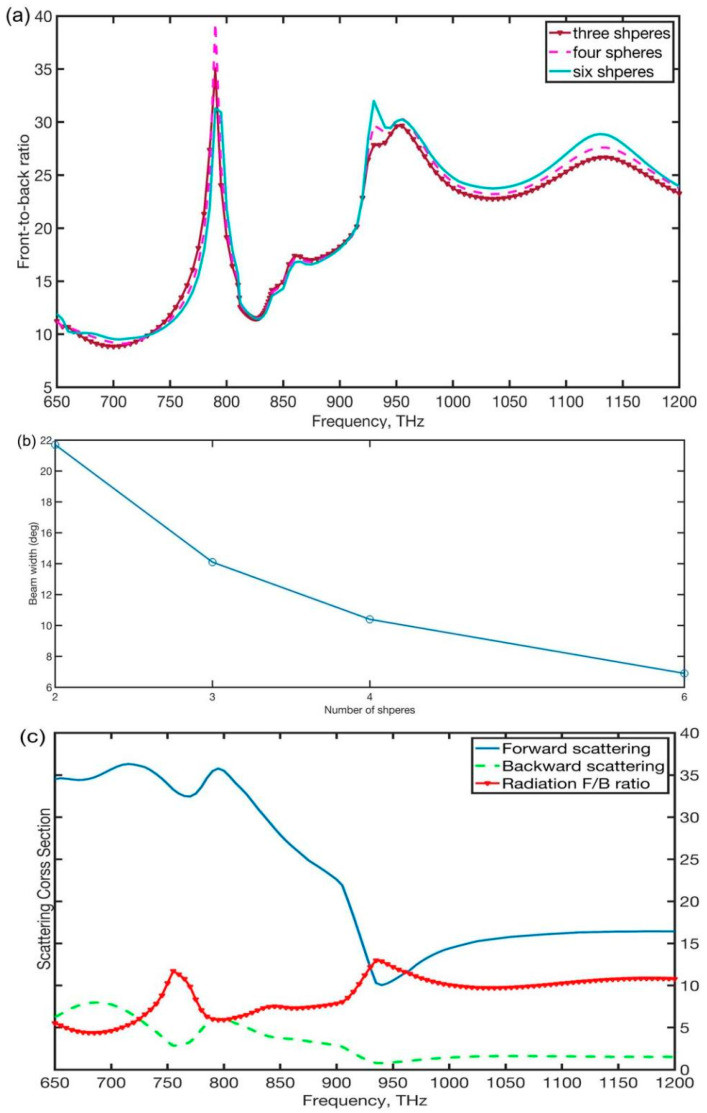
(**a**) the scattering F/B ratio and (**b**) beamwidth of the trimer, tetramer, and hexamer made up of MDM spheres arraying along the *y*-axis, (**c**) the scattering cross-section to the forward and backward direction, and the F/B ratio of the 230 nm diameter silver dimer.

We also show nanoantenna of the trimer, tetramer and hexamer made up of MDM spheres arraying along the *y*-axis (gap of spheres l = 0 nm). As shown in Figure 5a,b, under a plane wave with electric polarization perpendicular to the *y*-axis, the scattering front-to-back and beam with these structures of nanoantennas were observed in this section. In addition, they also demonstrate that these nanoantennas have a high F/B ratio (suppress 30) and can also achieve the first Kerker condition since the electric hexadecapole resonance in the particle was effectively excited at around 795 THz. Furthermore, compared to the hybrid dimer nanoantenna, as the number of nanospheres increases, the beamwidth of these coupled nanoantennas decrease obviously and the hexamer nanoantenna obtained an extremely narrow beamwidth of 6.9 deg. In addition, for the dimer of two silver spheres under a plane wave with electric polarization perpendicular to the dimer axis, the forward scattering cross-section and the scattering F/B ratio are calculated, as shown in Figure 5c. Compared to the hybrid dimer, the Ag dimer cannot achieve the Kerker condition at a frequency close to the peak of the dimer scattering spectrum due to mismatching in the amplitudes and phases of the electric and magnetic moments of the dimer.

## 4. Conclusions

In this contribution, we unambiguously indicated the mechanism of unidirectional radiation with high radiation efficiency through the symmetric dimer which is excited by an emitter, and strongly unidirectional forward scattering enhancement through the symmetric dimer under a light wave (incident light polarization parallel to the dimer axis) at optical frequency. The diameters of the metal and dielectric elements, as well as the gap spacing and position of an emitter, are physically and precisely tuned. The optimized symmetric MDM dimer manifests a high backward-to-forward radiation ratio (surpass 53 and almost attains the second Kerker condition) at 814 THz with a high radiation efficiency of 87% according to a flexible position of the emitter and the distance of the gap. In order to determine the relative contributions of specific electric and magnetic multipolar resonances, the multipole decomposition expansion is used to examine the dimer’s spectral responses. The symmetric dimer spheres (incident light polarization perpendicular to the dimer axis) sustained unidirectional forward scattering, which result in a nearly ideal Kerker condition in the frequency close to the resonance peak of the dimer due to enhancing the amplitude of the induced high-order electric multiples in the gap and effectively superimposing them with magnetic ones, which respond to the excitation of the plane wave in the dielectric layer of the dimer. In addition, we observed that the optimal MDM trimer, tetramer, and hexamer can also sustain unidirectional forward scattering with high scattering efficiency and extremely small beamwidth (below 6.9 deg). The flexibility in designing dimer geometry between metal and dielectric materials enables efficient unidirectional forward scattering and the controlling of the emitter radiation in a broadband spectrum, greatly expanding the application scope. The proposed hybrid MDM dimer nanoantenna might be used as a powerful and adaptable building block for controlling electromagnetic waves, which could be favorable in a variety of applications including metasurfaces, communications, and so on.

## Data Availability

Not applicable.
